# Supplementary material for: FANCD2 and RAD51 recombinase directly inhibit DNA2 nuclease at stalled replication forks and FANCD2 acts as a novel RAD51 mediator in strand exchange to promote genome stability
Source: Nucleic Acids Res. 2023 Aug 1;51(17):9144–65. doi: 10.1093/nar/gkad624 (PMC10516637; doi:10.1093/nar/gkad624)
Supplement: gkad624_Supplemental_File [file gkad624_supplemental_file.docx]

**SUPPORTING MATERIAL**

**Table S1 Reagents and materials**

| Antibodies | | | | | |
| --- | --- | --- | --- | --- | --- |
| Mouse monoclonal anti-MUS81 | | | | Abcam | ab14387 |
| Rabbit polyclonal anti-SMARCAL1 | | | | Bansbach et al., 2009 |  |
| Rabbit polyclonal anti-ZRANB3 | | | | Bethyl | A303-033A |
| Mouse monoclonal anti-GAPDH | | | | Millipore | MAB374 |
| Mouse monoclonal anti-BrdU (IdU) | | | | BD Biosciences | 347580 |
| Rat monoclonal anti-BrdU (CldU) | | | | Abcam | ab6326 |
| Mouse monoclonal anti-BrdU for IF | | | | Abcam | ab8152 |
| Goat anti-rat Alexa Fluor 594 | | | | Thermo Fisher | A-11007 |
| Goat anti-mouse Alexa Fluor 488 | | | | Thermo Fisher | A-11029 |
| Rabbit polyclonal anti-DNA2 | | | | Abcam | ab96488 |
| Mouse monoclonal anti-MRE11 | | | | GeneTex | GTX70212 |
| Mouse monoclonal anti-FANCD2 | | | | Santa Cruz biotechnology | sc-20022 |
| Rabbit polyclonal anti-SLX4 | | | | Bethyl | A302-269A-1 |
| Rabbit polyclonal anti-RAD51 | | | | Abcam | ab133534 |
| Phospho-Histone H2A.X (Ser139) | | | | Cell Signaling Technology | #2577 |
| Mouse monoclonal anti-GAPDH | | | | Millipore | AB2302 |
| RPA2 S33 phosphorylation | | | | Abcam | ab211877 |
| RPA2 T21 phosphorylation | | | | Abcam | ab109394 |
| Mouse monoclonal anti-FLAG | | | | Sigma | F1804 |
| Mouse monoclonal anti-RPA2 | | | | Abcam | Ab2175 |
| Mouse monoclonal anti-His tag | | | | Proteintech | 66005-1 |
| Rabbit polyclonal to Histone H3 | | | | Abcam | Ab1791 |
| Chemicals and reagents | | | |  |  |
| Hydroxyurea | | | | Sigma-Aldrich | H8627 |
| Camptothecin | | | | Sigma-Aldrich | PHL89593 |
| MRE11 protein | | | | Tanya Paull, UT Austin |  |
| EXO1 protein | | | | Paul Modrich, Duke University |  |
| RuvC | | | | Abcam | ab63828 |
| Recombinant human RAD51 | | | | Abcam | ab81943 |
| Cisplatin | | | | Sigma-Aldrich | 1134357 |
| Protease inhibitor cocktail | | | | Roche | 05892970001 |
| Genmute | | | | SignaGen | SL100568 |
| CldU | | | | Sigma-Aldrich | C6891-100MG |
| IdU | | | | Sigma-Aldrich | I7125-5G |
| Critical Commercial Assays | | | | | |
| Neutral COMET assay | | | | Trevigen | |
| Combing assay kit | | | | Genomic Vision | |
| Cell Lines | | | | | |
| Cell line | | | Source | | |
| U2OS | | | ATCC | | |
| HEK293T | | | ATCC | | |
| A549 | | | ATCC | | |
| BL21(DE3) CodonPlus | | | Agilent Technologies Cat#230280 | | |
| PD352i | | | FA Cell Repository at the Oregon Health & Science University | | |
| Plasmids | | | | | |
| pLKO.1 shSCR | | | Karanja et al., 2012 | | |
| pLKO.1 shDNA2 | | | Karanja et al., 2012 | | |
| pCMVΔR8.2 | | | Karanja et al., 2012 | | |
| pCMV-VSV-G | | | Karanja et al., 2012 | | |
| pET15b-FANCD2-His | | | Takahashi et al., 2014 | | |
| pCMV7.1-FLAG-DNA2 | | | Lin et al., 2013 | | |
| siRNA sequence | | | | | |
| Gene name | | Sequence | | | |
| SMARCAL1 | | | GCUUUGACCUUCUUAGCAA | | |
| ZRANB3 | | | GAUUCGAUCUAAUAACAGU | | |
| SLX4#2 | | | GAAGUGGAAUUGUCUAGCA | | |
| FANCD2 pool of 4 | | | UACCUCAAGUGUAUCCAUG | | |
|  |  |  | GGAGAUUGAUGGUCUACUA | | |
|  |  |  | CAACAUACCUCGACUCAUU | | |
|  |  |  | GGAUUUACCUGUGAUAAUA | | |
| BRCA2 | | | GAAACGGACUUGCUAUUUA | | |
| MRE11 | | | GCUAAUGACUCUGAUGAUATT | | |
| CTIP | | | GCUAAAACAGGAACGAAUCTT | | |
| RAD51#1 | | | CGAUGUGAAGAAAUUGGAATT | | |
| RAD51 J12 | | | Bhat et al., 2018 | | |
| DNA2 | | | ACAGUUGCCUGCAUUCUAA | | |
| FANCM | | | AGACAUCGCUGAAUUUAAA | | |
| MUS81 | | | CAUUAAGUGUGGGCGUCUA | | |
| Oligonucleotide substrates | | | | | |
| Name | Sequence | | | | |
| JYM945 | ACGGCATAAAGCTTGACGATTACATTGCTAGGACATCTTTGCCCACCTGCAG-GTTCACCC (131) | | | | |
| 87 FORK | TTCACGAGATTTACTTATTTCACTGCGGCTACATGATGCATCGTTAGGCGATT-CCGCCTAACGATGCATCATGTTGTTACCCTTTGA | | | | |
| LU 5’ FLAP | TTCACGAGATTTACTTATTTCACTGCGGCTACATGATGCATCGTTAGGCGATT-CCGCCTAACGATGCATCATGTCGCGAACCCTATTTAGGGTTCGCG | | | | |
| LU 3’  FLAP | CGCGAACCCTATTTAGGGTTCGCGACATGATGCATCGTTAGGCGATTCCGCC-TAACGATGCATCATGTTTCACGAGATTTACTTATTTCACTGCGGCT | | | | |
| Strand 1 | CGCTGCCGAATTCTACCAGTGCCATTGCTTTGCCCACCTGCAGGTTCACC | | | | |
| Strand 2 | GGTGAACCTGCAGGTGGGCAAAGCAATAGTAATCGTCAAGCTTTATGCCG | | | | |
| Strand 3 | CGGCATAAAGCTTGACGATTACTATTGGCTGTCTAGAGGATCCGACTATC | | | | |
| Strand 4 | GATAGTCGGATCCTCTAGACAGCCAATGGCACTGGTAGAATTCGGCAGCG | | | | |
| Strand 1L | TTCACGAGATTTACTTATTTCACTGCGGCTCGCTGCCGAATTCTACCAGTGC-CATTGCTTTGCCCACCTGCAGGTTCACC | | | | |
| JYM925 | GGGTGAACCTGCAGGTGGGCAAAGATGTCCTAGCAATGTAATCGTCAAGCTT-TATGCCGT (131) | | | | |
| EXO1: 3’ overhang | ACATGATGCATCGTTAGGCGATTCCGCCTAACGATGCATCATGTTTCACGAG-ATTTACTTATTTCACTGCGGCT | | | | |
| EXTJYM925 | CCTATGATCATTCCTTAAGGCTATCCTGAGTACCTCAGTCGGGTGAACCTGC-AGGTGGGCAAAGATGTCCTAGCAATGTAATCGTCAAGCTTTATGCCGT | | | | |
| RJ-167 | CTGCTTTATCAAGATAATTTTTCGACTCATCAGAAATATCCGTTTCCTATATTTATTCCTATTATGTTTTATTCATTTACTTATTCTTTATGTTCATTTTTTATATCCTTTACTTTATTTTCTCTGTTTATTCATTTACTTATTTTGTATTATCCTTATCTTATTTA (Jensen et al., 2010) | | | | |
| RJ-PHIX-42-1 | CGGATATTTCTGATGAGTCGAAAAATTATCTTGATAAAGCAG (Jensen et al., 2010) | | | | |
| RJ-PHIX-42-2 | TAATACAAAATAAGTAAATGAATAAACAGAGAAAATAAAGGC | | | | |
| RJ-Oligo1 | TAATACAAAATAAGTAAATGAATAAACAGAGAAAATAAAG (Jensen et al., 2010) | | | | |
| RJ-Oligo2 | CTTTATTTTCTCTGTTTATTCATTTACTTATTTTGTATTA (Jensen et al., 2010) | | | | |
| RJ-Oligo3 | AATTTTTCGACTCATCAGAAATATCCGTTTCCTATATTTA | | | | |
| RJ-Oligo4 | TAAATATAGGAAACGGATATTTCTGATGAGTCGAAAAATT | | | | |
| 3'Bio-RJ-PHIX-42-1 | CGGATATTTCTGATGAGTCGAAAAATTATCTTGATAAAGCAG/3Bio/ (Jensen et al., 2010) | | | | |
| Oligo#90 | CGGGTGTCGGGGCTGGCTTAACTATGCGGCATCAGAGCAGATTGTACTGAGAGTGCACCATATGCGGTGTGAAATACCGCACAGATGCGT (Jensen et al., 2010) | | | | |
| Oligo#60 | ACGCATCTGTGCGGTATTTCACACCGCATATGGTGCACTCTCAGTACAATCTGCTCTGATGCCGCATAGTTAAGCCAGCCCCGACACCCG (Jensen et al., 2010) | | | | |

**SUPPLEMENTAL FIGURES**

**
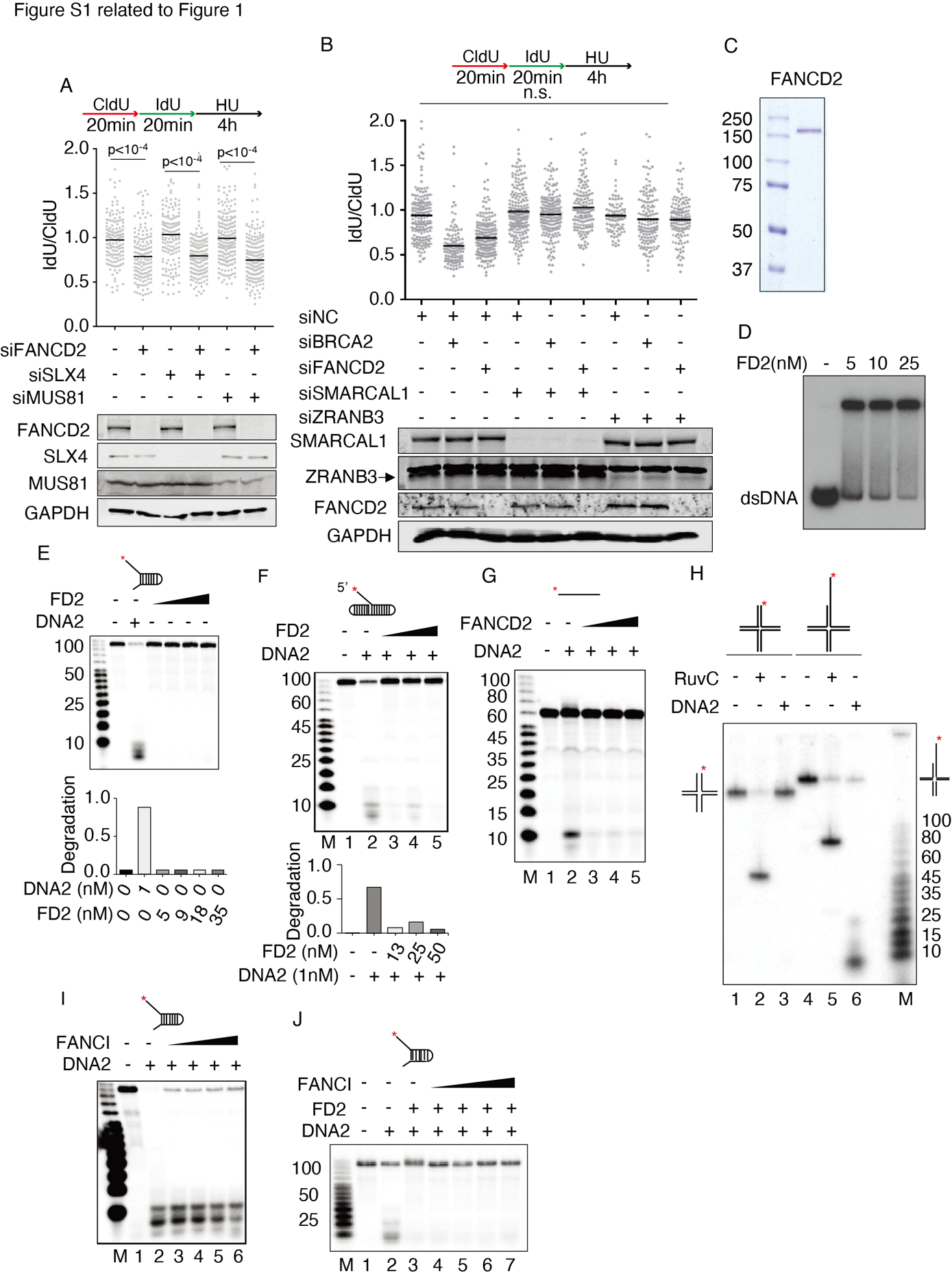
**

**Figure S1 (related to Figure 1). FANCD2-mediated Inhibition of in vivo and in vitro resection by DNA2**

(A-B) Over-resection of nascent DNA in HU-treated FANCD2 deficient cells is fork reversal dependent. U2OS cells were co-transfected with 12 nM siRNA for each indicated gene. 72 hours post-transfection, cells were pulsed by CldU and IdU, followed by 4 mM HU for 4h, as indicated on the top of the panel. The cells were harvested and analyzed by a DNA fiber assay. The IdU and CldU track lengths were measured, and the ratio was graphed (≥150 fibers were analyzed). A one-way ANOVA test was performed, n=2. Western blots show the level of knockdown in each panel.

(C) Human FANCD2-His was expressed and purified from insect cells as described (Roques et al., 2009). Gel analysis of the protein is shown.

(D) dsDNA binding by the FANCD2-His was carried out as described (Niraj et al., 2017).

(E) FANCD2 does not show nuclease activity under the conditions of DNA2 nuclease assay, as described in Materials and Methods. Markers show specific DNA2 nuclease product at 10 nt and smaller. Lane 1, DNA forked substrate without protein; lane 2, DNA2 alone; lanes 3-6, FANCD2 alone, the concentration is 5, 9, 18, 35 nM.

(F) FANCD2 inihibits DNA2 on a flap substrate. The assay was conducted as in Figure 1C, FANCD2 concentrations are 13, 25, 50 nM, respectively.

(G) FANCD2 inhibits DNA2 on ssDNA. with JYM945 substrate DNA (1.5 nM), FANCD2 concentrations are 13, 25,50nM, respectively.

(H) Oligonucleotides described in Table S1 were labeled, annealed and gel purified. The reversed fork with blunt ends consisted of 4 oligonucleotides: 5’ labeled strand 1, strand 2, strand 3, and strand 4 (van Gool et al., 1998). The reversed fork with 5’ overhang consisted of 5’ labeled strand 1L: strand 2, strand 3, and strand 4. 1.5 nM of the indicated substrate, 5’ end-labeled as indicated, was incubated in a 10 μl reaction mix containing 25 nM RuvC (Abcam) or diluent, 50 mM Tris-HCl (pH 8.0), 10 mM MgCl2, 100 μg/ mL BSA,1 mM DTT at 37°C for 30 min (Amunugama et al., 2018). Following incubations, proteinase K and SDS were added to 1 mg/ml and 0.5% respectively and incubated for 10 min at 37°C. 1 μl of 10X native loading dye was added to final 2.5% Ficoll- 400, 10 mM Tris-HCl (pH 7.5) and 0.0025% xylene cyanol concentrations. Samples were separated on an 8% native gel using 29:1 30% acrylamide solution, constant voltage 200V in cold room, 1X TAE, 120 min, 5 h exposure. The labeled product on the blunt ended reversed fork was expected to be 50 bp and 80 bp on the recessed reversed fork.

(I) FANCI does not inhibit DNA2. Reaction conditions were as in Figure 1C and FANCI was added at the concentrations indicated at the same time as DNA2.

(J) FANCI does not stimulate inhibition of DNA2 by FANCD2. Reaction conditions were as in Figure 1C and FANCI was added at the concentrations indicated at the same time as DNA2. FANCI concentrations are 8, 16, 32, 75 nM, respectively.

**
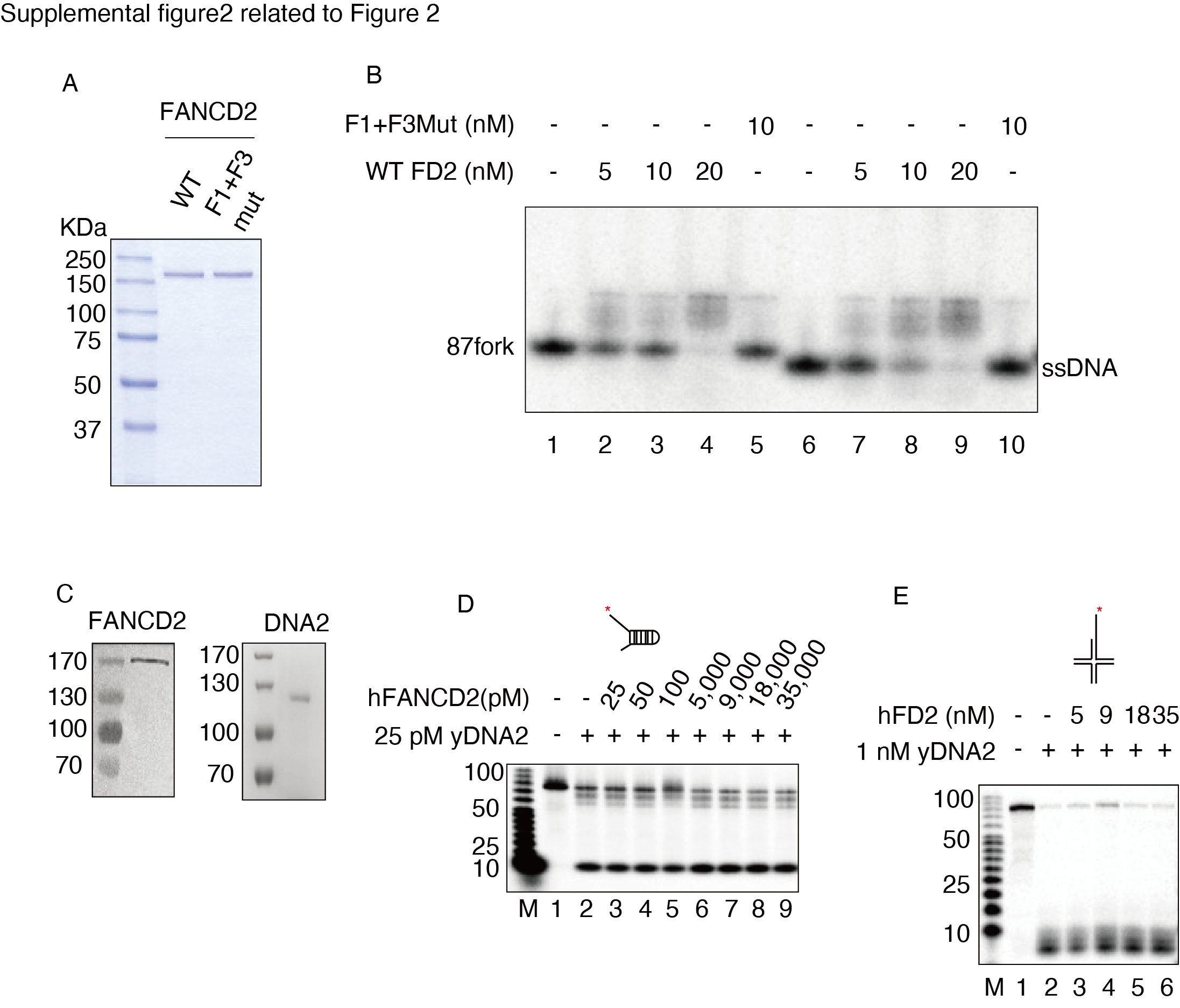
Figure S2 (related to Figure 2)**. **On the roles of FANCD2/DNA and FANCD2/DNA2 protein/protein reaction in the inhibition of DNA2 by FANCD2.**

(A) Human FANCD2 WT and FANCD2-F1+F3 Mut-His purification gel analysis.

(B) Comparison of DNA binding by FANCD2 and FANCD2-F1+F3Mut. Reaction conditions were as in (Niraj et al., 2017). Reactions in lanes 1-5 contained 1 nM 5’ labeled 87 FORK and reactions in lanes 6-10 contained 1 nM ssDNA: 5’ labeled JYM945* (60nt). Imagequant quantification revealed a 10- fold reduction in FANCD2-F1+F3Mut binding to ssDNA (lane8 vs lane 1).

(C) Purified FANCD2-His and FLAG-DNA2. Proteins were purified as described in Materials and Methods and analyzed by SDS gel.

(D) Yeast DNA2 is not inhibited by FANCD2 – 87 FORK substrate. Conditions were the same as in Figure 1D except that yeast FLAG- DNA2 (Masuda-Sasa et al., 2006) was used instead of human. Note that yeast DNA2 is more active than human DNA2 and therefore the amounts of yeast DNA2 shown are adjusted to give ≤ 50% degradation of substrate.

(E) Yeast DNA2 is not inhibited by FANCD2 – reversed fork substrate. Conditions were the same as in Figure 1E except that yeast FLAG-DNA2 (Masuda-Sasa et al., 2006) was used instead of human DNA2.

**
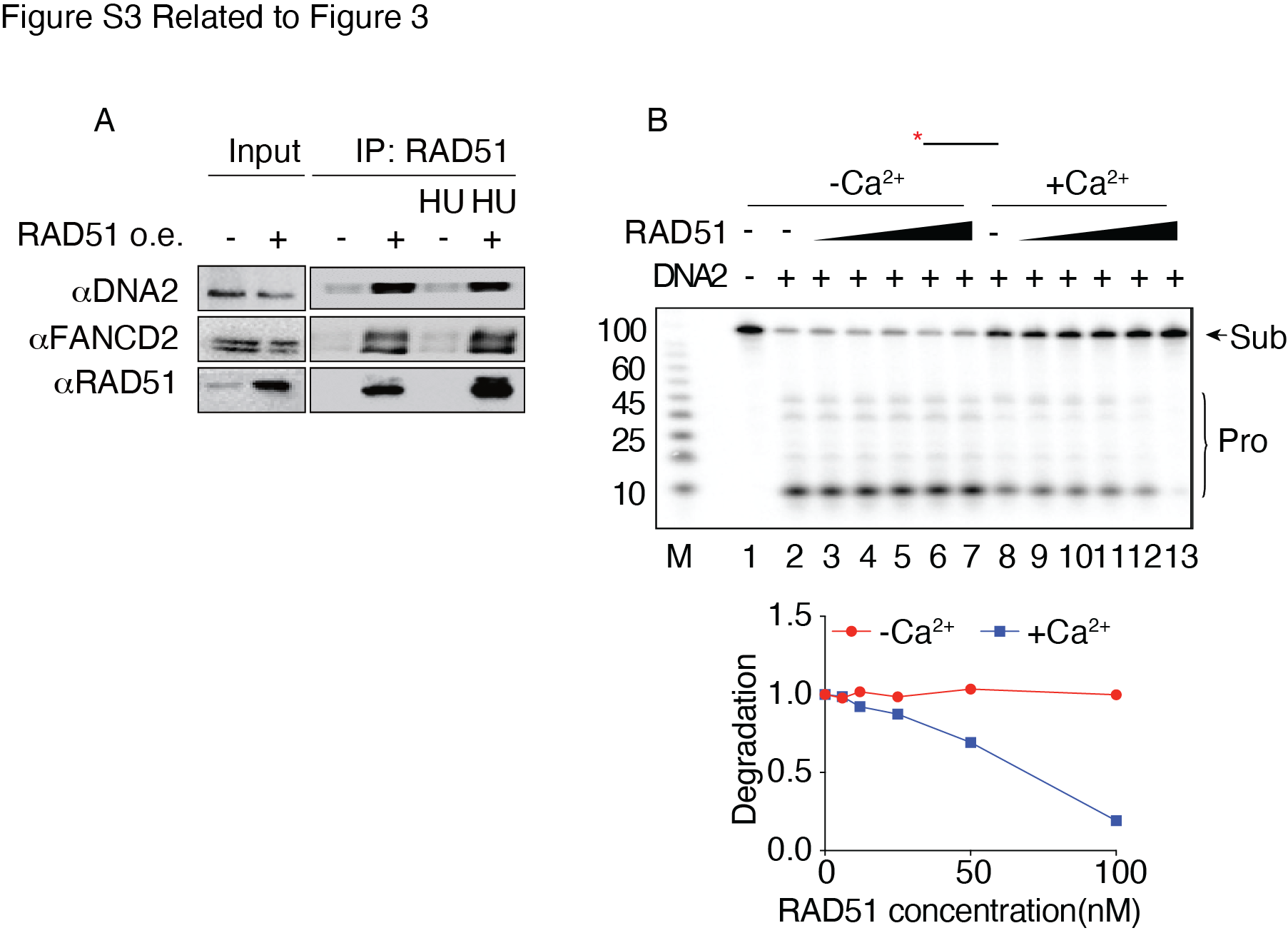
**

**Figure S3 (related to Figure 3). RAD51 filaments inhibit DNA2**.

(A) Co-immunoprecipitation of FANCD2 and RAD51 using RAD51 antibody for immunoprecipitation. RAD51 vector or empty vector was transfected into 293T cells and cells were split 24 hours post-transfection. At 48 hours post-transfection, cells were incubated with or without 2 mM HU for 3 hours; cells were then harvested and lysed. Lysates were incubated with RAD51 antibody and IgG-agarose beads, then washed with lysis buffer. Immunoprecipitates were analyzed for FANCD2 and DNA2 by western blotting on an 8% acrylamide gel.

(B) RAD51 filaments inhibit DNA2 on a ssDNA substrate. Increasing amounts of RAD51 were preincubated with 4 nM ssDNA (JYM945) in 25 mM TrisOAc (pH 7.5), 2 mM MgCl_2_, 2 mM ATP, 0.1 mg/ml BSA, and 2 mM DTT for 10 min at 37°C, conditions shown to be permissive for both filament formation and DNA2 nuclease activity in control assays (not shown). DNA2 was then added to 5 nM as indicated and reactions incubated for 30 min at 37°C. Samples were processed, run on a sequencing gel and analyzed by image J. Ca^2+^ (2 mM) was present in lanes 8-13.


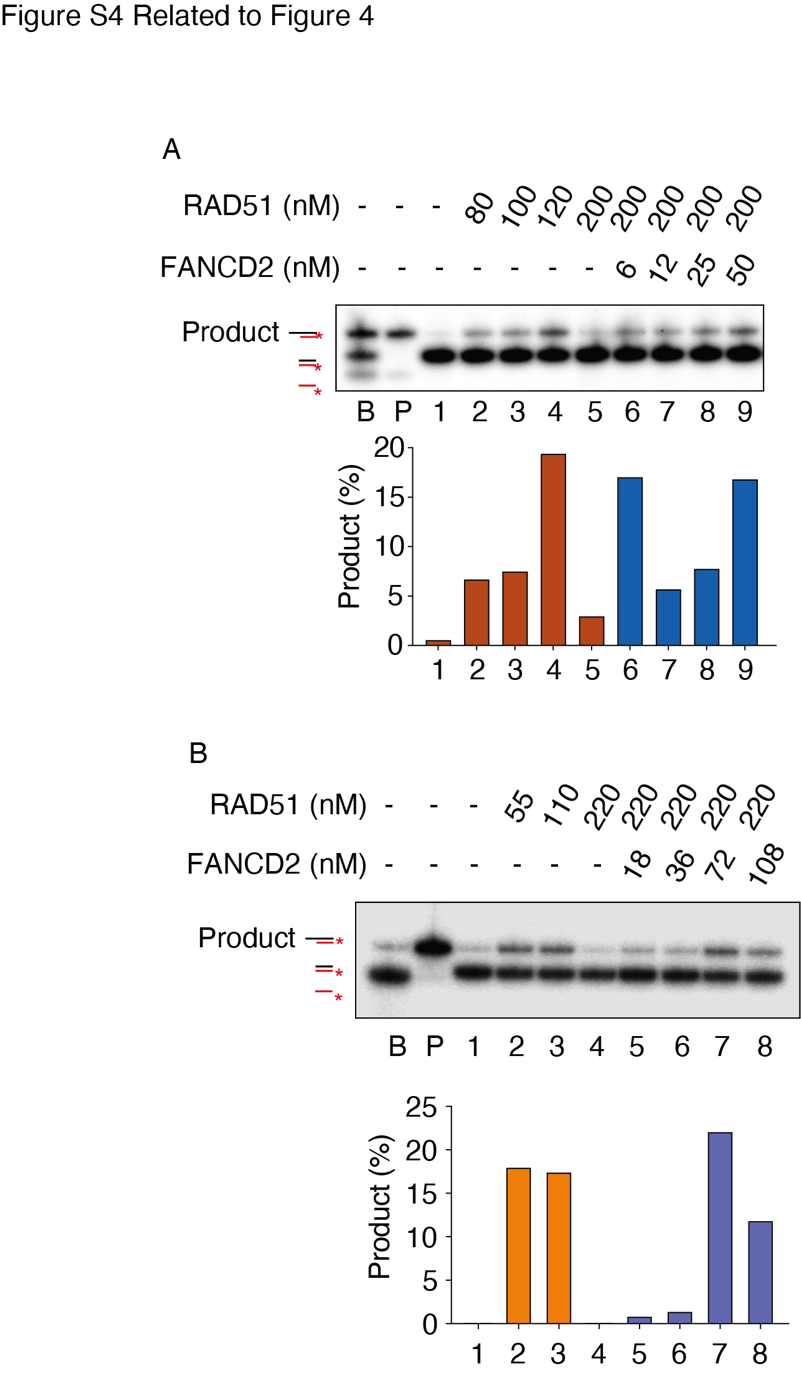


**Figure S4 (related to Figure 4). FANCD2 stimulates strand exchange on ssDNA by high levels of RAD51.** (A-B) Quantification is shown below each gel. Lane labeled B contains DNA markers for each relevant DNA species as indicated in the schematic on the left and was prepared by annealing oligonucleotide EXTJYM925, JYM925, and 5’ labeled JYM945; the lane labeled P is the marker for the position of the exchanged strand product (EXTJYM925 and 5’ labeled JYM945). A) Lanes 1-5: 4 nM ssDNA (100 nt, oligonucleotide EXTJYM925) was incubated with indicated amounts of RAD51 for 5 min at 37˚C and the 5’ labeled dsDNA (60mer, JYM925/JYM945 oligonucleotides) (final concentration 4 nM) was added and incubation continued for an additional 30 min at 37˚C for strand exchange. Lanes 6 to 9, RAD51 plus FANCD2 at the indicated concentrations present during both the 5’ preincubation with ssDNA and after the addition of dsDNA. The histogram below shows quantitation. B) Lanes 1-4: 4 nM ssDNA (100 nt, oligonucleotide EXTJYM925) was incubated with indicated amounts of RAD51 for 5 min at 37˚C and the 5’ labeled dsDNA (60mer, JYM925/JYM945 oligonucleotides) (final concentration 4 nM) was added and incubation continued for an additional 30 min at 37˚C for strand exchange. Lanes 5 to 8, RAD51 plus FANCD2 at the indicated concentrations present during both the 5’ preincubation with ssDNA and after the addition of dsDNA. The histogram below shows quantitation.


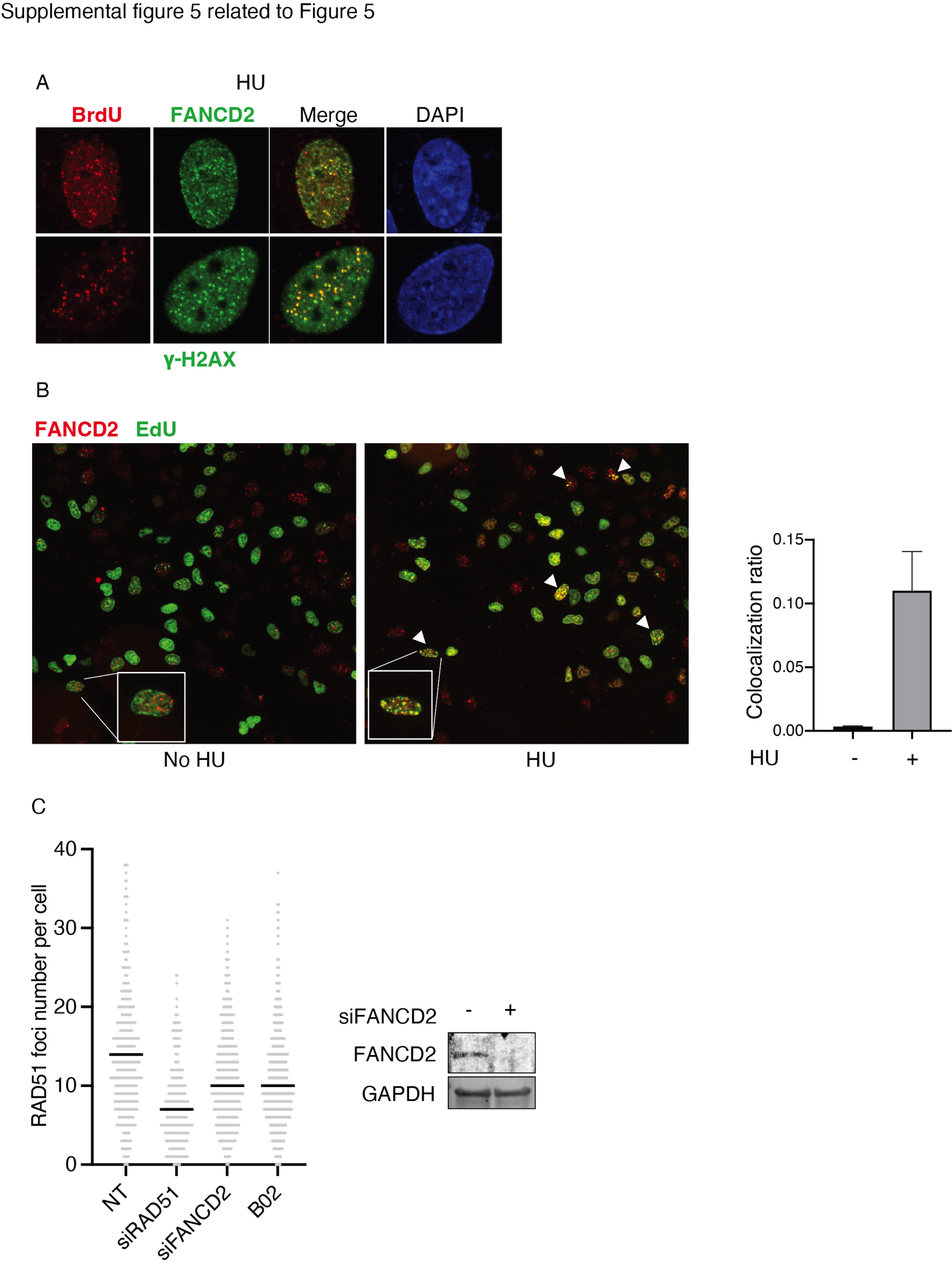


**Figure S5 (related to Figure 5). FANCD2 stabilizes RAD51 filaments at stalled fork**s.

(A) γH2AX and FANCD2 show co-localization with BrdU in response to HU. A549 cells were treated with 100 μM BrdU for 20 minutes, and then HU was added for 30 mins (Couch et al., 2013). Cells were prepared for immunofluorescence under native conditions as indicated in Method Details. Cells were stained with BrdU and γH2AX antibodies or BrdU and FANCD2 antibodies as indicated in the figure and imaged: anti-BrdU, red; α−γH2AX, green; α- FANCD2 green; DAPI, blue.

(B) FANCD2 co-localizes with nascent DNA after fork stalling. U2OS cells were labeled with 10 μM EdU for 10 minutes. After EdU labeling, 4 mM HU was added for 2 h. Then the U2OS cells were prepared for immunofluorescence as described in Method Details, together with non-HU treated cells. The number of cells with greater than 4 foci that showed colocalization of FANCD2 (red) and EdU (green) were counted, and the ratio of the number of cells with colocalized foci over total cells (including non-EdU stained cells) was determined and plotted. Over 800 cells were counted for each. Error was derived from standard deviation from two independent experiments.

(C) RAD51 foci are reduced in FANCD2 depleted cells. U2OS cells were transfected with 16 nM RAD51 or FANCD2 siRNA, respectively. 72 hours after transfection, the cells were treated with 4 mM HU with or without 25 μM RAD51 inhibitor B02 for 2 hours, then the cells were pre-extracted and fixed for immunofluorescence. Each data point represents the number of RAD51 foci in each cell. The bar represents the median of the total population. N=2.
